# The association of liver fat content and serum alanine aminotransferase with bone mineral density in middle-aged and elderly Chinese men and postmenopausal women

**DOI:** 10.1186/s12967-016-0766-3

**Published:** 2016-01-13

**Authors:** Ming-Feng Xia, Huan-Dong Lin, Hong-Mei Yan, Hua Bian, Xin-Xia Chang, Lin-Shan Zhang, Wan-Yuan He, Xin Gao

**Affiliations:** Department of Endocrinology and Metabolism, Zhongshan Hospital, Fudan University, Shanghai, China; Department of Ultrasonography, Zhongshan Hospital, Fudan University, Shanghai, China; Institute of Chronic Metabolic Diseases, Fudan University, Shanghai, China

**Keywords:** NAFLD, Bone mineral density, Liver fat content, Alanine aminotransferase, Osteocalcin

## Abstract

**Background:**

Recent studies have linked non-alcoholic fatty liver disease (NAFLD) to a reduced bone mineral density (BMD). We aimed to detect the quantitative association of liver fat content (LFC) and serum alanine aminotransferase (ALT) with BMD in a middle-aged and elderly Chinese population.

**Methods:**

The lumbar spine, hip and whole body BMDs were measured using dual-energy x-ray absorptiometry (Lunar iDXA, GE Healthcare) in 1659 Chinese (755 men and 1028 postmenopausal women) from Shanghai Changfeng community. Liver fat content was quantified via an ultrasound quantitative method. Multivariate linear regression analyses were carried out to determine the independent association of LFC and serum ALT with BMD and bone metabolic biomarkers. We also attempted to investigate the synergistic association between LFC and ALT as risk factors for bone mineral loss in Chinese.

**Results:**

Subjects with higher LFC had significantly lower BMD at all skeletal sites. Univariate correlation analysis showed that both LFC and ALT were inversely associated with BMD at the spine (r = −0.116, P < 0.001 and r = −0.102, P = 0.005), hip (r = −0.095, P = 0.014 and r = −0.075, P = 0.041) and whole body sites (r = −0.134, P < 0.001 and r = −0.164, P < 0.001) in men. After confounders were controlled for, LFC and ALT remained associated with BMD and bone formation biomarkers in men, but not postmenopausal women. When both NAFLD and elevation of ALT were present, there was a significant synergistic worsening of the BMDs at all bone sites.

**Conclusions:**

Liver fat content and serum ALT were inversely correlated with BMD in middle-aged and elderly men. The underlying mechanism might relate to a reduction in osteoblast activity. Elevation of the hepatotoxic biomarker ALT may indicate high risk for osteoporosis in patients with NAFLD.

**Electronic supplementary material:**

The online version of this article (doi:10.1186/s12967-016-0766-3) contains supplementary material, which is available to authorized users.

## Background

As the incidence of obesity reaches the epidemic level worldwide, non-alcoholic fatty liver disease (NAFLD) is also becoming a main public health concern [1, 2]. Once loaded with excessive triglyceride, liver could secrete a serial of pro/anti- inflammatory cytokines to disrupt the normal function of distant organs [3]. Several studies have demonstrated the associations of NAFLD with type 2 diabetes, chronic kidney disease, and cardiovascular diseases [4–6]. The cross-talks of NAFLD with other organs and its role in the pathogenesis of metabolic diseases are attracting extensive attention [7, 8].

Osteoporosis, characterized by decrease of body bone mineral density (BMD), is an important public health problem in the aging society [9]. Previous laboratory studies have shown a complex network of interaction among the adipose tissue, liver and bone [10, 11]. In parallel to the experimental studies, several recent case–control studies reported a significant decrease of BMD [12–14] and increase of osteoporotic fracture risk [15] in subjects with NAFLD. However, most studies in adults used common ultrasonography to diagnose NAFLD, which provided no information on the severity of liver steatosis. The associations of liver fat content (LFC) and hepatotoxic biomarker alanine aminotransferase (ALT) with BMD have not been fully studied, and the mechanisms underlying the association between NAFLD and bone mineral loss are still not clear.

In the current study, we measure the LFCs by a ultrasound quantitative method [16] in a large-scale middle-aged and elderly Chinese population, and investigated the association of LFC and ALT with BMD. Our study aimed to provide new insights into the relation between NAFLD and age-related bone mineral loss.

## Methods

### Study subjects

The present study consisted of 2224 participants of Shanghai Changfeng Study, a community-based prospective cohort study of chronic diseases in a middle-aged and elderly Chinese population, who were consecutively recruited from May 2010 to June 2011 [17]. Among the initial 2224 participants, 441 were excluded from study enrollment because they were individuals with viral hepatitis B or C (86 persons), renal failure (75 persons), history of thyroid dysfunction (17 persons), excessive alcohol consumption ≥10 g/d for women and ≥20 g/d for men (143 persons) [18], without BMD data (120 persons), and 124 premenopausal women were also excluded. Finally, a total of 1659 community participants (755 men and 904 postmenopausal women; age range 46–93 years; mean 63.2 ± 10.3 years) were included in the study. This study was approved by the Research Ethics Committee of the Shanghai Health Bureau, and all participants provided written informed consent.

### DXA measurements of BMD and body composition analysis

BMD (g/cm^2^) at the lumbar spine (L1–L4), total hips and the whole body region and the body fat masses at the whole body, trunk, and limbs sites were measured using dual-energy x-ray absorptiometry (Lunar iDXA, GE Healthcare). Body fat distribution was represented by the ratio of trunk to appendicular fat mass (trunk-appendicular fat ratio) [19]. A single, trained technician at a single clinical center carried out all measurements.

### Quantitative ultrasonography

Hepatic ultrasound examination was performed in all patients by an ultrasonographist (who was unaware of the clinical details of the participants). The ultrasound images were taken by a GE Logiq P5 scanner (GE Healthcare, Milwaukee, WI, USA), analyzed using NIH image software (ImageJ 1.41o, National Institutes of Health, Bethesda, MD, USA) and standardized using a tissue-mimicking phantom (Model 057; Computerized Imaging Reference Systems, Norfolk, VA, USA) to correct for the instrument differences. As detailed in our previous work [16], all the instrument settings, including “gain”, “depth”, and “time-gain compensation”, were calibrated using the tissue-mimicking phantom before measurement. The ultrasound images with both liver and right kidney clearly visualized in the sagittal liver/kidney view and the right liver lobe in right intercostal view at anterior axilla line were captured under the ultrasound machine and transferred to a personal computer installed with NIH image software. In the sagittal liver/right kidney view, a region of interest (ROI) of 1.5 × 1.5 cm (1296 pixels) in the liver parenchyma and another ROI of 0.5 × 0.5 cm (144 pixels) in the right renal cortex at the same depth was selected. In right intercostal view at anterior axilla line, two ROIs of 1.5 × 1.5 cm (1296 pixels) were also selected in liver homogeneous regions near the liver anterior margin (depth 4–6 cm) and the liver posterior margin, respectively. The echo intensity within the ROIs was measured, as well as the linear distance between the liver anterior and posterior ROIs. Then the LFC was obtained using the automatic calculator (Additional file 1) based on the following equation: LFC (%) = 62.592 × ultrasound hepatic/renal ratio + 168.076 × hepatic attenuation rate −27.863 [16]. The LFC by ultrasound quantitative method showed excellent correlation and agreement with that by proton magnetic resonance spectroscopy previously (r = 0.89, P < 0.001), and this method has been used to investigate the associations of LFC with carotid atherosclerosis and diabetes previously [20, 21]. The subjects were defined as NAFLD if their LFCs by quantitative ultrasonography ≥9.15 % [16, 21].

### Anthropometric and biochemical measurements

Each participant underwent a clinical examination that consisted of an interview by a trained investigator, anthropometric measurements and serum biochemical examinations. Information regarding demographics, lifestyle, medical history and current medication was obtained through interview and questionnaires. The questionnaire regarding alcohol intake included items about the type of alcoholic beverage consumed, the frequency of alcohol consumption on a weekly basis and the usual amount of daily alcohol consumption. Participants were classified as non- or light-drinkers or as excessive drinkers when their average alcohol consumption was <140 g/week for men (<70 g/week for women) or >140 g/week for men (>70 g/week for women) [18], respectively. Smoking status was defined as non-smoker or smoker. Standing height and bodyweight were measured without shoes or outer clothing. The body mass index (BMI) was calculated as weight (kg) divided by height squared (m^2^). Blood samples were obtained after a fasting period of at least 12 h. Total cholesterol (TC), high-density lipoprotein cholesterol (HDL-c), low-density lipoprotein cholesterol (LDL-c), triglycerides (TG), creatine, uric acid and liver enzymes were measured using a model 7600 automated bio-analyzer (Hitachi, Tokyo, Japan). The fasting blood glucose (FBG) and 2 h post load glucose levels following a 75 g oral glucose challenge for non-diabetics were measured using the glucose oxidase method. Serum osteocalcin, 25-hydroxyvitamin D [25(OH)D] and β-isomerized form carboxy-terminal telopeptide of type I collagen (β-CTx) were measured using electrochemiluminescence immunoassay. The glomerular filtration rate (eGFR) was calculated using the Modification of Diet in Renal Disease (MDRD) Study formula [22].

### Statistical analysis

All statistical analyses were performed using SPSS software version 15.0 (SPSS, Chicago, IL, USA). Kolmogorov–Smirnov Test was used for test of normality. Data were presented as mean ± SD, except for skewed variables, which were presented as median with the interquartile range (25–75 %) given in parentheses. Anova or the Kruskal–Wallis test was used for intergroup comparisons of continuous data, whereas the Chi squared test was used for comparisons of categorical variables. We stratified all analyses by sex [23], and the skewed variables were log transformed to approximate normal distribution before analyses. Correlations of BMD with LFC, serum ALT as well as other metabolic parameters were investigated using the linear correlation analysis after adjustment for age and body weight [24–26]. Multiple linear regression models were used to examine the independent associations of LFC with spine, hip and whole body BMD, adjusting for age, body weight, total body fat percentage (BF %), trunk-appendicular fat ratio and the variables that displayed significant associations with BMD in the univariate correlation analysis. We also calculated the sample size for the multivariate linear regression analysis, and a sample size of 1184 would allow for the detection of small effects (r^2^ = 0.02) in multiple regression analysis with all covariates in the analysis with 2-tailed alpha of 0.05 and power of 0.80 [27].

To determine if ALT levels and LFC had synergistic effects on age-related bone mineral loss, the average BMDs were compared across three groups: (1) subjects without NAFLD; (2) subjects with NAFLD and normal ALT (≤40 IU/L); (3) subjects with NAFLD and increased ALT level (>40 IU/L). All P values reported were two-tailed and P < 0.05 was considered significant.

## Results

### Clinical characteristics of the study population

The prevalence of NAFLD, metabolic syndrome and diabetes in the study population was 31.8, 29.2 and 18.7 %, respectively. The median LFC for the 1659 subjects was 4.9 % (interquartile range, 2.1–11.5 %). Subjects were stratified into four subgroups according to the quartiles of LFC, and subjects with higher LFC were younger, more obese and had lower BMD at all bone sites. Higher LFC was in parallel with higher BF %, trunk fat mass, appendicular fat mass and trunk-appendicular fat ratio, and correlated with higher liver enzymes, blood pressure, fbg and unfavourable lipid profiles (All P < 0.05). Serum levels of osteocalcin were also significantly decreased in subjects with elevation of LFC (Table 1).

**Table 1:** Demographic characteristics and metabolism and bone status of the study population

	Total	Liver fat content				P value
		Q1	Q2	Q3	Q4	
Demographic						
Number (men/women)	1659 (755/904)	413 (192/221)	416 (194/222)	414 (185/229)	416 (184/232)	0.859
Age (years)	62 (56–72)	63 (56–74)	64 (57–74)	61 (55–71)^a,b^	62 (55–70)^a,b^	0.005
Alcohol drinking n (%)	128 (7.2 %)	30 (7.3 %)	29 (7.0 %)	36 (9.2 %)	30 (7.2 %)	0.606
Cigarette smoking n (%)	340 (19.1 %)	76 (18.4 %)	85 (20.4 %)	90 (21.7 %)	84 (20.2 %)	0.694
Height (cm)	161.4 ± 8.3	161.4 ± 8.0	161.0 ± 8.0	161.5 ± 8.4	161.8 ± 8.4	0.677
Weight (kg)	63.4 ± 10.8	60.8 ± 9.4	60.8 ± 10.1	63.0 ± 10.7^a,b^	68.9 ± 10.7^a,b,c^	<0.001
BMI (kg/m^2^)	24.3 ± 3.3	23.3 ± 2.7	23.4 ± 3.0	24.1 ± 3.4^a,b^	26.3 ± 3.1^a,b,c^	<0.001
Medication						
Calcium n (%)	215 (12.1 %)	57 (13.8 %)	51 (12.3 %)	43 (10.4 %)	4 9 (11.8 %)	0.508
Vitamin D n (%)	92 (5.2 %)	28 (6.8 %)	19 (4.6 %)	15 (3.6 %)	22 (5.3 %)	0.206
Bisphophonates n (%)	4 (0.2 %)	0 (0.0 %)	0 (0.0 %)	1 (0.2 %)	2 (0.5 %)	0.301
Metabolic parameters						
SBP (mmHg)	134 (123–150)	133 (121–147)	133 (121–148)	133 (122–149)	137 (128–153)^a,b,c^	<0.001
DBP (mmHg)	77 (70–83)	76 (69–83)	75 (68–82)	76 (69–83)	79 (73–85)^a,b,c^	<0.001
ALT (IU/L)	16 (12–22)	15 (12–19)	14 (11–19)	16 (12–21)	20 (14–28)^a,b,c^	<0.001
AST (IU/L)	20 (17–23)	20 (17–22)	19 (17–22)	19 (17–23)	20 (18–25)^a,b,c^	0.001
GGT (IU/L)	24 (18–36)	21 (16–29)	21 (17–31)	24 (18–38)^a,b^	29 (21–42)^a,b,c^	<0.001
Triglyceride (mmol/L)	1.4 (1.1–2.1)	1.3 (1.0–1.7)	1.3 (1.0–1.8)	1.5 (1.1–2.1)^a,b^	1.8 (1.3–2.5)^a,b,c^	<0.001
Cholesterol (mmol/L)	5.12 ± 0.95	5.13 ± 0.93	5.02 ± 0.91	5.16 ± 0.94^b^	5.16 ± 1.00^b^	0.130
HDL-c (mmol/L)	1.4 (1.1–1.6)	1.5 (1.2–1.7)	1.4 (1.2–1.7)	1.3 (1.1–1.6)^a,b^	1.2 (1.1–1.4)^a,b,c^	<0.001
LDL-c (mmol/L)	2.95 ± 0.81	3.00 ± 0.79	2.87 ± 0.76^a^	2.97 ± 0.81	2.94 ± 0.86	0.133
Apo-A (U/L)	1.4 (1.3–1.6)	1.52 (1.32–1.70)	1.44 (1.26–1.66)^a^	1.41 (1.24–1.62)^a^	1.37 (1.21–1.56)^a,b,c^	<0.001
Apo-B (U/L)	1.02 ± 0.22	1.00 ± 0.21	0.98 ± 0.23	1.03 ± 0.21	1.06 ± 0.22^a,b,c^	<0.001
FBG (mmol/L)	5.1 (4.8–5.7)	5.0 (4.7–5.6)	5.1 (4.8–5.6)	5.1 (4.8–5.6)	5.3 (4.9–6.0)^a,b,c^	<0.001
PBG (mmol/L)	6.8 (5.6–8.8)	6.3 (5.3–7.9)	6.4 (5.3–7.9)	6.8 (5.6–9.2)^a,b^	7.8 (6.6–10.2)^a,b,c^	<0.001
eGFR (mL/min)	95 (84–108)	95 (83–110)	94 (83–106)	95 (85–110)	94 (83–108)	0.134
Body composition						
Body fat percentage (%)	34.2 (28.9–39.1)	32.7 (27.6–38.2)	32.9 (27.8–37.7)	34.3 (28.8–39.0)^a,b^	36.4 (31.6–41.2)^a,b,c^	<0.001
Trunk fat (kg)	11.6 (9.3–14.0)	10.5 (8.5–12.7)	10.6 (8.2–12.8)	11.5 (9.5–13.9)^a,b^	13.8 (11.6–16.2)^a,b,c^	<0.001
Appendicular fat (kg)	7.5 (6.2–9.1)	7.0 (5.8–8.6)	7.2 (5.9–8.6)	7.4 (6.1–8.9)^a^	8.4 (7.0–10.0)^a,b,c^	<0.001
Trunk-Appendicular fat ratio	1.50 (1.29–1.72)	1.44 (1.24–1.63)	1.40 (1.20–1.64)	1.54 (1.32–1.76)^a,b^	1.62 (1.45–1.83)^a,b,c^	<0.001
Liver fat content (%)	4.9 (2.1–11.5)	0.5 (0.0–1.4)	3.4 (2.8–4.1)^a^	7.5 (6.1–9.3)^a,b^	16.6 (14.1–21.4)^a,b,c^	<0.001
Bone status						
Spine BMD (g/cm^2^)^d^	1.0624 ± 0.1683	1.0715 ± 0.1703	1.0728 ± 0.1627	1.0693 ± 0.1708	1.0359 ± 0.1674^a,b,c^	0.004
Hip BMD (g/cm^2^)^d^	0.8174 ± 0.1163	0.8216 ± 0.1180	0.8238 ± 0.1156	0.8199 ± 0.1166	0.8043 ± 0.1144^a,b,c^	0.066
Whole-body BMD (g/cm^2^)^d^	1.0708 ± 0.1151	1.0811 ± 0.1193	1.0785 ± 0.1069	1.0717 ± 0.1171	1.0516 ± 0.1149^a,b,c^	<0.001
Osteocalcin (ng/ml)	18.6 (14.4–23.5)	19.0 (14.6–23.8)	19.8 (15.0–24.2)	18.6 (14.2–24.1)^b^	17.4 (13.7–22.0)^a,b,c^	<0.001
25(OH)Vit D (nmol/L)	44.5 (34.6–58.3)	46.5 (35.0–59.5)	42.9 (33.3–56.4)	43.7 (34.7–58.4)	44.9 (34.6–58.8)	0.339
ALP (U/L)	73 (62–87)	73 (62–87)	73 (62–87)	73 (61–86)	76 (64–90)	0.227
β-CTx (ng/ml)	0.40 (0.26–0.58)	0.39 (0.29–0.56)	0.40 (0.27–0.61)	0.41 (0.25–0.56)	0.40 (0.26–0.59)	0.500

*SBP* systolic blood pressure, *DBP* diastolic blood pressure, *ALT* alanine aminotransferase, *AST* aspartate aminotransferase, *GGT* gamma-glutamyl transferase, *Apo-A* apolipoprotein A, *Apo-B* apolipoprotein B, *FBG* fasting blood glucose, *PBG* postprandial blood glucose, *GFR* glomerular filtration rate, *BMD* bone mineral density, *25(OH)VitD* 25-hydroxyvitamin D, *β-CTx* β-isomerized form carboxy-terminal telopeptide of type I collagen

^a^P < 0.05 compared with Q1

^b^P < 0.05 compared with Q2

^c^P < 0.05 compared with Q3

^d^Age and body weight were adjusted for before intergroup comparison

### Association of LFC and serum ALT level with BMD

**Fig. 1:**
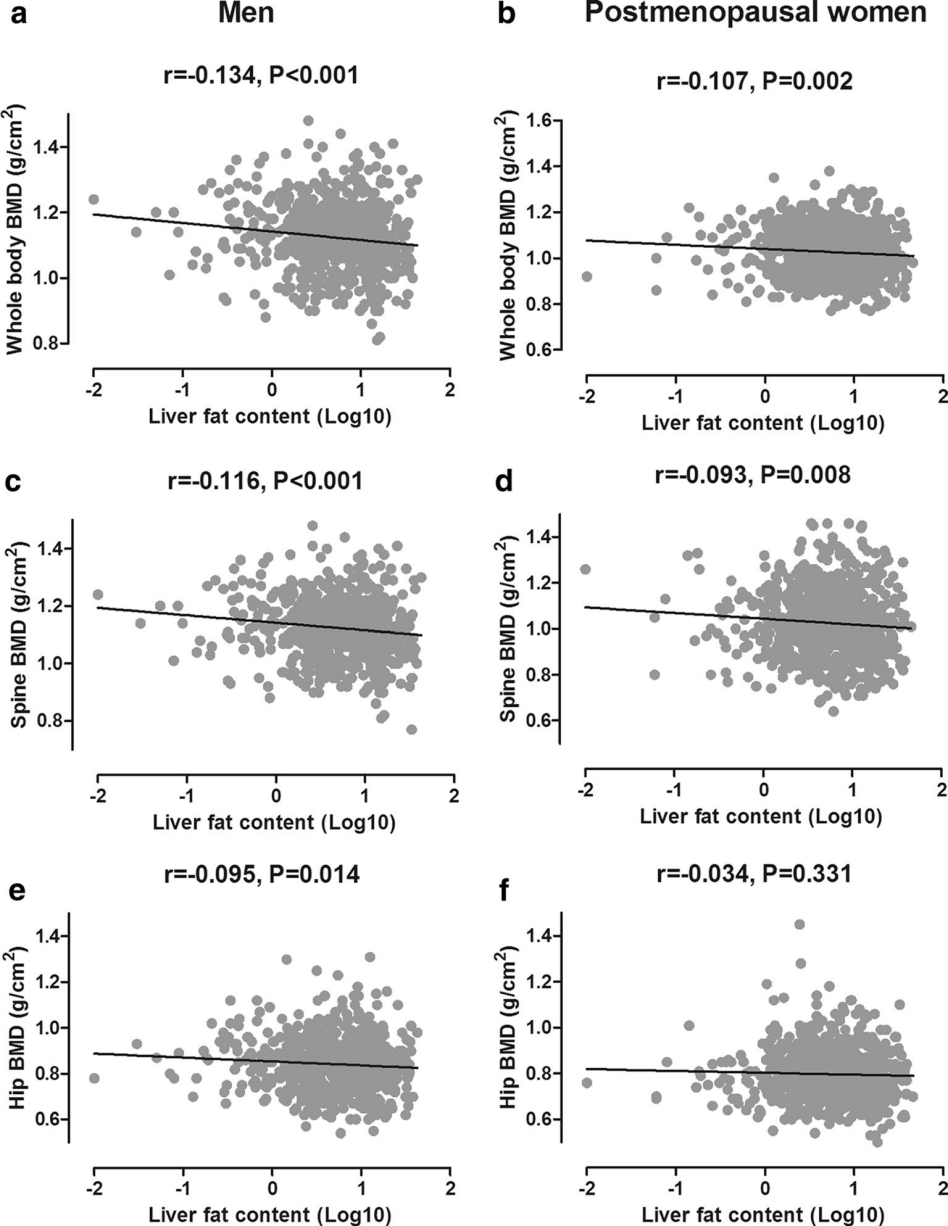
The associations between LFC and BMDs at the whole body (**a, b**), lumbar spine (**c, d**) and total hip (**e, f**) sites in middle-aged and elderly men (*panels on the left*) and postmenopausal women (*panels on the right*)

After age and body weight were controlled for, LFC was negatively associated with BMD in both men and women (Fig. 1). These associations were significant at lumbar spine (r = −0.116, P < 0.001), hip (r = −0.095, P = 0.014) and whole body site (r = −0.134, P < 0.001) in men. In postmenopausal women, significant negative associations were also observed at the whole-body site (r = −0.107, P = 0.002) and the lumbar spine (r = −0.093, P = 0.008).

**Fig. 2:**
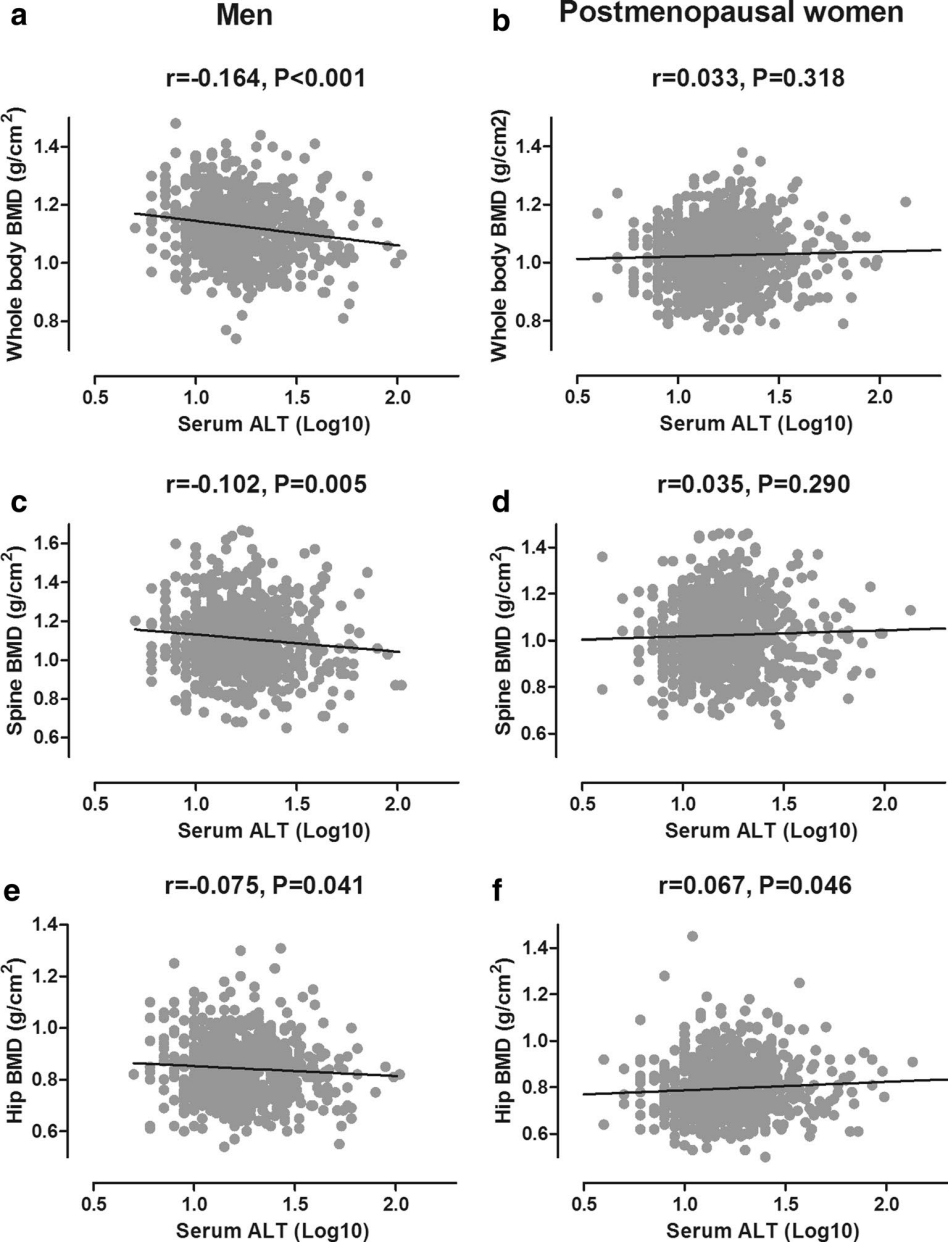
The associations between serum ALT level and BMDs at the whole body (**a, b**), lumbar spine (**c, d**) and total hip (**e, f**) sites in middle-aged and elderly men (*panels on the left*) and postmenopausal women (*panels on the right*)

Serum ALT is commonly measured clinically as a biomarker for liver injury. In men, there were also inverse correlations of serum ALT with BMD at the whole body (r = −0.164, P < 0.001), lumbar spine (r = −0.102, P = 0.005), and hip (r = −0.075, P = 0.041). In postmenopausal women, ALT showed no association with spine and whole body BMD, and only marginal significant association with hip BMD (r = 0.067, P = 0.046) (Fig. 2).

### Association of body fat distribution and metabolic parameters with BMD

In parallel to the change of LFC, increased total BF % was also significantly associated with lower BMD at the hip (r = −0.082, P = 0.025) and whole-body (r = −0.171, P < 0.001) in men, and BMD at all bone sites (r = −0.146 to −0.265, All P < 0.001) in women. Trunk-appendicular fat ratio represented the central distribution of body fat, and showed negative correlation with whole-body (r = −0.122, P < 0.001) and hip BMD (r = −0.069, P = 0.027) in postmenopausal women and whole-body BMD (r = −0.078, P = 0.034) in men (Table 2).

**Table 2:** Association of bone mineral density with liver fat content and metabolic parameters

	Spine BMD		Hip BMD		Whole body BMD	
	r	P	r	P	r	P
Men						
Cigerette smoking	−0.186	<0.001	−0.095	0.009	−0.158	<0.001
Alcohol drinking	−0.004	0.914	−0.020	0.584	−0.043	0.241
Liver fat content	−0.116	<0.001	−0.095	0.014	−0.134	<0.001
Body fat percentage	0.019	0.600	−0.082	0.025	−0.171	<0.001
Trunk to appendicular fat ratio	0.058	0.119	0.005	0.884	−0.078	0.034
SBP	−0.178	<0.001	0.057	0.125	0.063	0.086
FBG	0.070	0.058	0.041	0.264	−0.008	0.817
Triglyceride	−0.019	0.616	−0.030	0.418	−0.112	0.002
Cholesterol	−0.025	0.504	−0.126	0.001	−0.098	0.007
HDL-c	0.037	0.322	−0.026	0.476	0.048	0.196
Uric acid	0.022	0.543	0.021	0.559	−0.007	0.842
Anti-osteoporotic drug	0.001	0.999	−0.015	0.679	−0.018	0.616
Postmenopausal women						
Cigerette smoking	−0.064	0.057	−0.010	0.771	0.001	0.992
Alcohol drinking	−0.025	0.446	0.033	0.326	−0.013	0.700
Liver fat content	−0.093	0.008	−0.034	0.331	−0.107	0.002
Body fat percentage	−0.127	<0.001	−0.119	<0.001	−0.227	<0.001
Trunk to appendicular fat ratio	−0.069	0.027	−0.045	0.152	−0.112	<0.001
SBP	−0.099	0.003	−0.108	0.001	−0.163	<0.001
FBG	−0.067	0.046	−0.025	0.464	−0.107	0.001
Triglyceride	0.000	0.999	0.001	0.994	−0.034	0.307
Cholesterol	−0.013	0.688	0.001	0.992	−0.010	0.764
HDL-c	−0.042	0.206	−0.009	0.791	−0.004	0.911
Uric acid	0.136	<0.001	0.080	0.045	0.105	0.002
Anti-osteoporotic drug	0.029	0.386	0.007	0.895	0.023	0.494

Age and body weight were adjusted

*SBP* systolic blood pressure, *FBG* fasting blood glucose, *HDL-c* high-density lipoprotein-cholesterol

Significant associations between BMD and NAFLD-related metabolic parameters were also found in both men and postmenopausal women. BMDs at specific bone site were significantly associated with systolic blood pressure (SBP), serum TG and TC in men, and SBP, FBG and uric acid in postmenopausal women after adjustment for age and body weight. Cigarette smoking is also related to decreased BMD at all bone sites in men (Table 2).

### Independent contribution of LFC and ALT to variability in BMD and bone metabolic biomarkers

In multiple regression analysis, after adjustment for age and body weight (Table 3,4 model 1), LFC and serum ALT were significantly correlated with BMD at the lumbar spine (Stdβ = −0.116, P = 0.003 and Stdβ = −0.102, P = 0.005), total hip (Stdβ = −0.097, P = 0.014 and Stdβ = −0.075, P = 0.041) and whole-body BMD (Stdβ = −0.134, P < 0.001 and Stdβ = −0.164, P < 0.001) in men. LFC was also negatively associated with whole-body (Stdβ = −0.107, P = 0.002) and spine (Stdβ = −0.093, P = 0.008) BMD in postmenopausal women. Parameter estimates of these correlations remained significant after successively adjusting for use of osteoporotic drug, alcohol drinking, cigarette smoking, and metabolic parameters such as SBP, TG, TC, FBG, uric acid in both genders (model 2) and then in addition total BF % and trunk-appendicular fat ratio in men (model 3). In model 4, the significant negative correlation between LFC and BMD remained at all bone sites after additional adjustment for serum 25(OH)D, osteocalcin and β-CTx level (model 4). In multiple regression models, LFC and ALT was also negatively associated with serum osteocalcin in men (models 1–3).

**Table 3:** Multiple linear regression analysis for the association between liver fat content (independent variable) and BMD and bone metabolic parameters (dependent variables) in different models

	Model 1		Model 2		Model 3		Model 4	
	Std β	P	Std β	P	Std β	P	Std β	P
Men								
Lumbar spine BMD	−0.116	0.003	−0.111	0.003	−0.111	0.003	−0.123	0.001
Hip BMD	−0.095	0.014	−0.101	0.007	−0.092	0.017	−0.101	0.008
Whole-body BMD	−0.134	<0.001	−0.122	0.001	−0.123	0.001	−0.130	<0.001
25(OH)D	−0.073	0.044	−0.027	0.457	−0.018	0.638	–	–
Osteocalcin	−0.183	<0.001	−0.133	<0.001	−0.116	0.001	–	–
β-CTx	0.014	0.715	0.015	0.694	0.023	0.560	–	–
Postmenopausal women								
Lumbar spine BMD	−0.093	0.008	−0.086	0.015	−0.056	0.116	−0.068	0.068
Hip BMD	−0.034	0.331	−0.044	0.215	−0.015	0.689	−0.029	0.429
Whole-body BMD	−0.107	0.002	−0.072	0.041	−0.023	0.510	−0.041	0.224
25(OH)D	0.027	0.410	0.034	0.332	0.044	0.223	–	–
Osteocalcin	−0.060	0.099	−0.046	0.197	−0.025	0.498	–	–
β-CTx	−0.035	0.298	−0.029	0.384	−0.021	0.563	–	–

Model 1 included liver fat content, age and body weight

Model 2 included liver fat content, age, body weight, alcohol drinking, cigarrete smoking, anti-osteporotic drug use, SBP, FBG, triglyceride, cholesterol, and uric acid

Model 3 included liver fat content, age, body weight, alcohol drinking, cigarrete smoking, SBP, FBG, triglyceride, cholesterol, HDL-c,uric acid, body fat percentage, and trunk to appendicular fat ratio

Model 4 included same as model 3 plus serum 25(OH)D, osteocalcin, and β-CTx levels

*Std β* denotes standardized β coefficient, *BMD* bone mineral density, *25(OH)VitD* 25-hydroxyvitamin D, *β-CTx* β-isomerized form carboxy-terminal telopeptide of type I collagen, *SBP* systolic blood pressure, *FBG* fasting blood glucose, *HDL-c* high-density lipoprotein-cholesterol

**Table 4:** Multiple linear regression analysis for the association between ALT (independent variable) and BMD and bone metabolic parameters (dependent variables) in different models (Men)

	Model 1		Model 2		Model 3		Model 4	
	Std β	P	Std β	P	Std β	P	Std β	P
Men								
Lumbar spine BMD	−0.102	0.005	−0.120	0.001	−0.120	0.001	−0.142	<0.001
Hip BMD	−0.075	0.041	−0.062	0.105	−0.056	0.151	−0.073	0.046
Whole-body BMD	−0.164	<0.001	−0.152	<0.001	−0.118	0.001	−0.143	<0.001
25(OH)D	0.012	0.747	0.017	0.647	−0.018	0.638	–	–
Osteocalcin	−0.093	0.012	−0.085	0.019	−0.102	0.006	–	–
β-CTX	0.001	0.994	0.001	0.981	0.002	0.952	–	–

Model 1 included liver fat content, age and body weight

Model 2 included liver fat content, age, body weight, alcohol drinking, cigarrete smoking, anti-osteporotic drug use, SBP, FBG, triglyceride, cholesterol, and uric acid

Model 3 included liver fat content, age, body weight, alcohol drinking, cigarrete smoking, SBP, FBG, triglyceride, cholesterol, HDL-c,uric acid, body fat percentage, and trunk to appendicular fat ratio

Model 4 included same as model 3 plus serum 25(OH)D, osteocalcin, andβ-CTx levels

*Std β* denotes standardized β coefficient, *BMD* bone mineral density, *25(OH)VitD* 25-hydroxyvitamin D, *β-CTx* β-isomerized form carboxy-terminal telopeptide of type I collagen, *SBP* systolic blood pressure, *FBG* fasting blood glucose, *HDL-c* high-density lipoprotein-cholesterol

### Interactions between ALT and LFC as risk factors for bone mineral loss in men

**Fig. 3:**
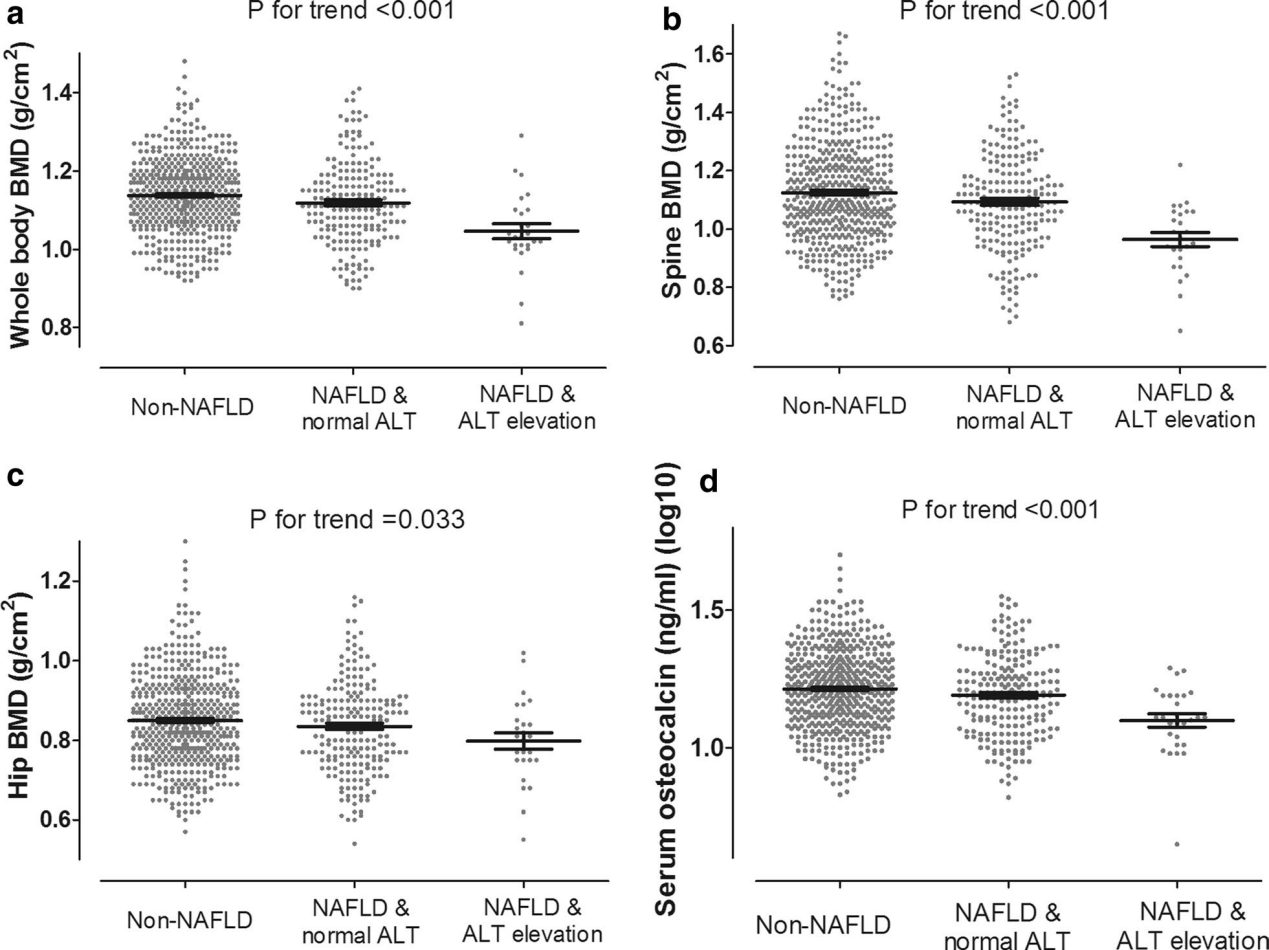
Comparison of average bone mineral density at lumbar spine, hip and whole body sites and serum osteocalcin level in subjects with normal LFC, NAFLD and ALT within the normal range, and NAFLD and ALT elevation. There was a synergistic decrease in BMDs and osteocalcin level in individuals with both NAFLD and ALT elevation

Compared to individuals with normal LFC, NAFLD patients had significantly lower BMD at the lumbar spine and whole body sites (Fig. 3a, b). When both NAFLD and elevation of ALT level were present, there was a synergistic worsening of the BMD at all bone sites, as shown in Fig. 3. Serum osteocalcin was also decreased with elevated serum ALT and presence of NAFLD.

## Discussion

In the current study, we provide the epidemiological evidence showing that LFC and serum ALT have independent and inverse relationships with BMD at the lumbar spine, hip and whole body sites in middle-aged and elderly Chinese men. Both LFC and ALT were associated with serum osteocalcin, a biochemical marker for the activity of osteoblasts in patients with NAFLD [28]. According to our results, elevation of the hepatotoxic biomarker ALT may indicate high risk for osteoporosis in patients with NAFLD.

Visceral fat accumulation has been related to decreased BMD [29, 30]. In accordance with the association between visceral fat and BMD, metabolic syndrome, featured by central obesity and insulin resistance, was demonstrated as a risk factor for developing osteoporosis clinically [31]. NAFLD is thought to be the hepatic manifestation of metabolic syndrome [1]. Therefore, a relationship between NAFLD and osteoporosis has been assumed. Several recent observations of lower BMD in obese children and postmenopausal women with NAFLD were reported [12, 13]. In the current study, using the ultrasound quantitative method for LFC [16], we found LFC was negatively and independently associated with BMD in middle-aged and elderly Chinese. This finding remained after controlling for total BF % and its central distribution. Therefore, liver fat might play a role in the pathogenesis of osteoporosis independent of centrally located body fat.

The mechanisms underlying the relation between NAFLD and low BMD are still not clear. We measured bone metabolism biomarkers in subjects with NAFLD, and found that serum osteocalcin, a well-established biomarker for osteoblast acitivity [28], was significantly decreased in male subjects with NAFLD, but the bone resorption markers (i.e., β-CTx) [32] remained unchanged. Therefore, the decrease of BMD in subjects with NAFLD might be related to an inhibition of osteoblast activities. There have been several hypotheses regarding pathogenesis of osteoporosis in NAFLD. Hepatic insulin resistance has been related to low circulating osteocalcin, decreased bone formation and deficient numbers of osteoblasts [33], and might be responsible for lower osteocalcin level and BMD in the NAFLD patients. However, the associations between LFC and BMD at all bone sites still existed after adjusting for all components of metabolic syndrome as well as the total body fat mass, so a direct interaction between liver tissue and bone [11] may also contribute to the accelerated bone loss in patients with NAFLD. As is shown previously, fat-infiltrated liver itself may produce a series of cytokines and other bone-influencing molecules, including TNFα, interleukin-6, interleukin-1 and Fetuin-A [34], and these molecules could further inhibit activity of osteoblast and osteogenesis [35]. Several previous studies also reported that NAFLD was related to vitamin D metabolism [36]. Vitamin D exerts a significant effect on calcium and bone metabolism, and was converted to 25(OH)D in the liver for its active form [37]. However, in our current study, 25(OH)D was not changed with increasing LFC. The inconsistency might be explained by the difference of liver function status. Most of NAFLD patients in our study had mild liver steatosis with normal liver enzyme levels, while the previous case–control study enrolled NAFLD patients mostly with elevated liver enzymes, who were more likely to have impaired function of vitamin D metabolism.

ALT is commonly measured clinically as a biomarker for hepatocellular injury. In the current study, ALT was also found to be associated with BMD in subjects with NAFLD. It has been demonstrated that ALT levels not only correlated with the severity of liver steatosis grades [38], but also associated with markers of systemic inflammation (CRP) [39] and oxidative stress [40] in NAFLD patients. The mechanisms of inflammation and oxidative stress were involved in the pathogenesis of age-related bone loss [41, 42]. Therefore, ALT can be used as a clinical biomarker for risk of osteoporosis in NAFLD patients.

We reported a relationship of LFC and serum ALT with BMD in middle-aged and elderly men, but not in women. The gender difference in the association between LFC and BMD has also been noticed in recent studies [14, 15]. The differences between men and women in features of age-related osteoporosis [43, 44], body fat deposition [45] and sex hormone levels could be potential explanations. However, we cannot deduce the mechanisms of the gender difference in our current study, which need to be elucidated further.

There are several limitations in this study. Firstly, the present study was a cross-sectional study, and did not permit the identification of causal relationships between LFC and BMD, which needs to be further evaluated in longitudinal studies. Secondly, in a study of our current size, it was not possible to obtain liver biopsies to investigate the association of BMD with liver fibrosis or inflammation. Thirdly, the sex hormone levels of the community subjects were not measured, so the effect of estrogen deficiency may interfere with the association between LFC and BMD in postmenopausal women.

## Conclusion

Osteoporosis is one of the major public health problems in the aging Chinese society. Our study has provided the epidemiological evidence that both LFC and hepatotoxic biomarker ALT were inversely correlated with BMD and biomarkers of bone formation in middle-aged and elderly Chinese men. Elevation of ALT, a traditional serum biomarker for hepatocellular injury, may indicate high risk for osteoporosis in patients with NAFLD.

## Additional file

10.1186/s12967-016-0766-3Program for liver fat content calculation based on ultrasonography quantitative method.

## Abbreviations

NAFLD: non-alcoholic fatty liver disease
BMD: bone mineral density
LFC: liver fat content
ALT: alanine aminotransferase
DXA: dual-energy x-ray absorptiometry
ROI: region of interest
BMI: body mass index
TC: total cholesterol
HDL-c: high-density lipoprotein-cholesterol
LDL-c: low-density lipoprotein-cholesterol
TG: triglycerides
FBG: fasting blood glucose
25(OH)D: 25-hydroxyvitamin D
β-CTx: β-isomerized form carboxy-terminal telopeptide of type I collagen
GFR: glomerular filtration rate
MDRD: modification of Diet in Renal Disease
BF %: body fat percentage
SBP: systolic blood pressure
